# Therapeutic strategies for primary heart involvement in systemic sclerosis

**DOI:** 10.1515/rir-2024-0010

**Published:** 2024-07-15

**Authors:** Veronica Batani, Lorenzo Dagna, Giacomo De Luca

**Affiliations:** Vita-Salute San Raffaele University, Milan, Italy; Unit of Immunology, Rheumatology, Allergy and Rare Diseases, IRCCS San Raffaele Hospital, Milan, Italy

**Keywords:** systemic sclerosis, scleroderma, primary heart involvement, heart, myocarditis, myocardial fibrosis, therapeutic strategies, therapy

## Abstract

Primary heart involvement (pHI) is frequent in systemic sclerosis (SSc), even though often underdiagnosed. SSc-pHI has been recently defined as cardiac abnormalities that are predominantly attributable to SSc rather than other causes and/or complications. SSc-pHI represents a major determinant of mortality in SSc, accounting alone for about 12% of disease-related deaths; its early recognition and promptly therapeutic interventions are therefore crucial. Both perfusion defects and myocardial inflammation contribute to the occurrence of myocardial fibrosis that precipitates myocardial remodeling, potentially leading to heart failure and arrhythmic complications. To date, clear evidence and guidelines for effectively managing SSc pHI are not established yet, resulting in a lack of a defined therapeutic algorithm. In this review we summarize the most recent scientific literature on the prevailing therapeutic strategies and interventions to manage SSc-pHI, with particular focus on therapeutic strategies to counteract the 3 major pathogenic events of the disease, *i.e*. microvascular damage, myocardial inflammation and myocardial fibrosis.

## Introduction

Primary heart involvement (pHI) is a major determinant of mortality in systemic sclerosis (SSc), accounting alone for about 12% of disease-related deaths. The high mortality of SSc-pHI is due to various reasons.^[1,2]^ Firstly, definite diagnostic algorithms are lacking and mainly based on expert opinion, and SSc-pHI still remains often unrecognized or misdiagnosed. As a consequence, its exact prevalence is unclear, ranging between 7 and 39%. Furthermore, SSc-pHI has been variously defined over the years, and only recently the 2022 consensus-based defined SSc-pHI as “cardiac abnormalities that are predominantly attributable to SSc rather than other causes and/or complications of the disease”.^[1]^

Secondly, both difficulties in distinguish primary from secondary heart involvement in SSc and the different sensitivity of diagnostic tools used clearly affect the possibility to timely detect SSc-pHI and to precisely estimate its prevalence.^[1]^

Then, the pathogenesis of SSc-pHI remains elusive. Both myocardial inflammation and perfusion defects contribute to the final occurrence of myocardial fibrosis, either focal or interstitial, historically considered the hallmark of scleroderma heart disease, and eventually leading to the development of late stage cardiac complications. Perfusion defects include both microvascular damage and aberrant vasoreactivity as potential contributors to myocardial pathology. Ischemia-reperfusion damage is postulated to initially precipitate contraction band necrosis, subsequently culminating in irreversible myocardial fibrosis.^[3]^ Recent evidences have highlighted that myocardial inflammation is frequent and often subtle in SSc patients and contribute to myocardial interstitial fibrosis, through the so-called “inflammation-driven pathway to fibrosis”.^[4,5,6,7]^

Myocardial fibrosis occurs therefore as the consequence of an intricate interplay between vascular and inflammatory mechanisms.^[8]^ Initial microvascular injury may represent the incipient pathogenic event, precipitating ischemia and subsequent endothelial activation, which precedes all subsequent pathological alterations. Autoimmune and inflammatory responses to cellular damage ultimately trigger fibroblast activation and their trans-differentiation into myofibroblasts, the principal architects of extracellular matrix protein synthesis, thereby fostering myocardial fibrosis.^[9,10]^ Macrophages have gained recognition for their pivotal role in myocardial fibrosis through interactions with fibroblasts, thus emerging as compelling therapeutic targets in SSc-associated cardiac disease.^[11]^ These cells are implicated in myocardial remodeling and diastolic dysfunction, with peripheral blood analyses of SSc patients revealing augmented expression of alternatively activated (M2) macrophages.^[12]^

The constellation of ischemic, inflammatory and fibrotic lesions, ensuing from the aforementioned pathophysiological processes, precipitates myocardial remodeling, eventually leading to heart failure (HF).^[13]^ Such alterations may represent, moreover, the ideal substrate for arrhythmias and conduction defects, which represents another major determinant of mortality, responsible for the dramatic occurrence of sudden cardiac death (SCD).^[6,14]^

Thus, within this dual-faceted ischemic-inflammatory pathogenic model, the interplay between reperfusion byproducts and pro-inflammatory cytokines orchestrates SSc-related cardiac involvement, furnishing a biological rationale for targeted therapeutic interventions.

Finally, the lack of definite therapeutic strategies to curb myocardial damage in SSc patients clearly contribute to the high mortality associated with SSc-pHI.

Although there is heightened interest from the healthcare community, clear evidence and guidelines for effectively managing SSc-pHI are not established yet, resulting in a lack of a unified approach to treatment, which are not reported neither in 2017^[15]^ nor in the newly revised recommendations by European League against Rheumatism (EULAR) for the treatment of SSc.^[16]^

Here we comprehensively reviewed the most recent scientific literature on SSc-pHI and the prevailing therapies to manage SSc-pHI. We focused the attention on therapeutic strategies to counteract the 3 major pathogenic events of the disease, *i.e*. microvascular damage, myocardial inflammation and myocardial fibrosis.

## Methods

The PubMed database was explored from 1964 to March 2024. Search terms included a combination of Medical Subject Headings (MeSH) and keywords related to SSc such as “Scleroderma, Systemic,” “Systemic sclerosis,” or “SSc,” along with cardiology-related terms like “primary heart,” “heart,” “cardiac,” “myocardial,” and “myocarditis,” in conjunction with “therapy” or “treatment.” Articles were initially screened by title and abstract, with those of relevance undergoing full-text review. The selection was limited to English-language articles specifically concerning SSc-pHI. Additionally, references from review articles were examined, and relevant titles not previously included were added to the reference pool. The results were then reported divided on the basis of the pathogenetic mechanism addressed by the drugs investigated.

## Drugs targeting myocardial inflammation

### Corticosteroids

Corticosteroid therapy has proven efficacious in the management of pericarditis and endomyocardial biopsy (EMB)-proven virus-negative myocarditis (VNM),^[7]^ and has therefore been tested for the treatment of SSc-related myocarditis.

In an open-label pilot study among SSc patients with myocarditis by Pussadhamma *et al*., 12 patients were treated with a dose equivalent to prednisolone 0.5 mg/kg/d for 2-weeks, then tapered off 10 mg every 2 weeks until 10 mg/d at week 4, then slowly tapered off completely by 24-weeks. At the end of treatment, 8 out of the 12 patients experienced a clinical improvement of myocarditis, while a proportion of patients died due to cardiac complications during treatment, particularly those with high high-sensitive cardiac troponin T (hs-cTnT), high N-terminal pro-brain natriuretic peptide (NT-proBNP), and impaired left-ventricular ejection fraction (LVEF).^[17]^

Other case reports and case series have previously explored the use of prednisone for SSc myocarditis, with negative results. Kerr *et al*.^[18]^ reported the use of prednisone in 6 patients with diffuse SSc who presented with concomitant myositis and myocarditis. Of these, four patients were treated solely with high-dose steroids as first-line therapy and all died for congestive HF.

Similarly, despite initially favorable responses, 2 out of 3 SSc patients with myositis and myocarditis treated with daily prednisone (40–100 mg) reported by West *et al*.^[19]^ developed conduction defects.

Evidences for the use of methylprednisolone (mPDN) monotherapy for SSc-myocarditis are also limited to case reports, with not conclusive results. Carette *et al*.^[20]^ used high-dose pulse mPDN to treat a patient with aggressive myocarditis and myositis refractory to prednisone, who had a therapeutic response with a more intensive regimen. A resolution of myocardial edema after treatment with pulse mPDN (1 g/ m^2^ i.v. for 3 consecutive days, followed by 20 mg i.m. for 30 days, then maintained at 4 mg/day) was also described in an asymptomatic SSc patient. ^[21]^ Conversely, Clemson *et al*.^[22]^ reported the failure of high-dose intravenous mPDN followed by a course of prednisone, with a fatal outcome due to progressive HF in an SSc patient.

Steroids have been more commonly associated with conventional immunosuppressants, mainly methotrexate, cyclophosphamide or azathioprine. Chiefly, Panoupolos *et al*. reported 2 cases treated with prednisone and methotrexate with functional improvement in one case (both left and right ventricular EF) and reduction of T1 and T2-mapping at cardiac magnetic resonance (CMR) in the other one.^[23]^

Thus, considering the paucity of data and the conflicting results (Table 1), the use of steroids in SSc-pHI could not be clearly recommended. Moreover, in SSc patients presenting with diffuse cutaneous involvement or rapid disease progression and in those seropositive for anti-RNA polymerase-III antibodies, glucocorticoids should be prescribed judiciously due to an elevated risk of precipitating scleroderma renal crisis.^[24,25]^

**Table 1:** Drugs targeting myocardial inflammation in SSc-pHI.

Drug	Type of study	N° patients	Diagnosis	Results
Cyclophosphamide	Case report, Stack, 2009^[26]^	1	Myocarditis (clinical) in SSc	Resolution of symptoms, normalization of cardiac enzyme and improvement of left ventricular function.
	Observational cohort study, Pieroni, 2014^[8]^	7 (4 treated with cyclophosphamide)	EMB-proven myocarditis in SSc	At 12-month follow-up significant clinical and laboratory improvement.
	Retrospective study Panopoulos, 2022^[23]^	18 (11 treated with cyclophosphamide)	CMR(2018 revised Lake Louise criteria)	Various degrees of effectiveness
Azathioprine	Observational cohort study Pieroni, 2014^[8]^	7 (3 treated immediately with AZA)	EMB-proven myocarditis in SSc	At 12-month follow-up significant clinical and laboratory improvement.
	Case reports Kerr 1994^[18]^ De Luca 2017^[6]^	3	Myocarditis (clinical) in SSc	Clinical and haemodynamic status improvement, normalization of cardiac enzyme.
	Prospective study Mavrogeni 2017^[29]^	7	Myocarditis (CMR)	Normalization of CMR alterations at 6 months
Mycophenolate	Prospective cohort study, De Luca, 2020^[30]^	2	EMB-proven myocarditis in SSc	6 months improvement of clinical status, LVEF on echocardiography, LV volumes and wall motion abnormalities.
	Case reports De Luca 2017^[6]^, 2018^[4]^	2	EMB-proven myocarditis in SSc	Clinical and biochemical improvement.
	Case series, Campochiaro 2019^[34]^	4 (3 patients treated with MMF and one patient was started on azathioprine)	Myocarditis in SSc	Two patients were achieved an optimal disease control.
	Retrospective study Panopoulos 2022^[23]^	18 (1 treated with mycophenplate)	CMR(2018 revised Lake Louise criteria)	Improvement at Holter ECG, LVEF, RVEF, T2 and T1 mapping, T2 ratio, ECV and LGE at CMR
Rituximab	Case series, Campochiaro 2019^[34]^	4 (only one patient treated with RTX as rescue therapy)	Myocarditis (clinical) in SSc	Only partial benefit.
	Retrospective study Panopoulos 2022^[23]^	18 (1 treated with CYC plus RTX)	CMR(2018 revised Lake Louise criteria)	favorable results on both RVEF, LVEF and T2 mapping, while no results on T1 mapping and ECV
Tocilizumab	Case series Campochiaro, 2019^[44]^	1	Cardiac Magnetic Resonance	Normalization of cardiac biomarkers and complete reversal of cardiac inflammation on CMR.
	Retrospective study Panopoulos 2022^[23]^	18 (1 treated with tocilizumab)	CMR(2018 revised Lake Louise criteria)	No significant result on cardiac enzymes, CMR features and contractile function.
	Case series Lee *et al*.^[45,46]^	2	CMR (LGE areas, elevated native T1 values and ECV).	CMR indices improvement, LVEF improvement
	Case report Ishizaki *et al*.^[45]^	1	EMB	Improvement of myocardial BMIPP uptake; LVEF 45% to 59 %
Intravenous immunoglobulins	Case report Cacciatore, 2018^[36]^	1 patient	Myocarditis (clinical) in SSc	progressive improvement of dyspnea and of heart systolic ejection fraction

SSC-pHI, systemic sclerosis-primary heart involvement.

### Disease-modifying Antirheumatic Drugs (DMARDs)

Reports on response to cyclophosphamide in the treatment of SSc-myocarditis are limited to case reports, case series and observational cohort study.

Stack *et al*. firstly described a case of myocarditis in a rapidly progressive diffuse cutaneous SSc successfully treated with a total of 12 pulsed intravenous (IV) cyclophosphamide (10 mg/kg monthly) and methylprednisolone (0.5 mg/kg given for 3 days each month with a maintenance dose of prednisolone 70 mg once daily between cycles), before receiving mycophenolate mofetil (MMF) and prednisolone 20 mg once daily as maintenance therapy. This therapeutic strategy resulted in resolution of patient’s symptoms and left ventricular function improvement at echocardiogram.^[26]^

In the more recent study by Panopoulus *et al*., 11 SSc patients with myocarditis received cyclophosphamide, mainly oral (7 cases); of these, 8 received cyclophosphamide as monotherapy, 3 combined with steroids and 1 combined with rituximab. An improvement of right-ventricular EF (RVEF) and LVEF was reported in 4 and 2 cases, respectively; considering CMR features at follow-up, T2-mapping values decreased in 5 cases and % of late gadolinium enhancement (LGE) was reduced in 4 cases, while a reduction in T1-mapping, T2 ratio and percentage extracellular volume (ECV) was observed in only one case each. The improvement of arrhythmic burden at 24 h-electrocardiogram (ECG) Holter was detected in a single case, and in 2 patients the functional and imaging improvement was paralleled by reduction of cardiac enzymes. To date, a significant clinical worsening of SSc-pHI was reported in 2 patients.^[23]^

In the pioneering study by Pieroni *et al*., 4 patients with newly diagnosed SSc-myocarditis were treated with high-dose intravenous glucocorticoid (betametasone 12 mg/day for 3 days, 8 mg/day for 3 days, and 6 mg/day for 3 days), followed by oral steroids (prednisone 0.5 mg/kg/day, gradually tapered) and cyclophosphamide (2 mg/kg/day up to a cumulative dose of 6 g) then followed by azathioprine. Patients who received cyclophosphamide as induction therapy simultaneously had a diffuse skin involvement or evidence of interstitial lung disease (ILD). After 12-months, all patients showed a significant clinical and laboratory improvement, although only one patient experienced a complete recovery of contractile function. Immunosuppressive therapy with steroids and cyclophosphamide was shown to be more effective in patients with lower degrees of fibrosis at EMB, suggesting that an early introduction of immunosuppressive agents is crucial to positively impact on SSc-pHI.^[8]^

Although associated with various degrees of effectiveness (Table 1), cyclophosphamide is noted for its low therapeutic profile and high toxicity, including cardiotoxicity.^[27]^ Although the cardiotoxicity of cyclophosphamide is dose dependent and related to higher doses than those used in rheumatology (cyclophosphamide > 1.55 g/m^2^/d in the context of bone marrow transplantation) its use in SSc-pHI should still be considered with caution.

The most robust data supporting the use of azathioprine in SSc myocarditis indirectly derives from the tailored iMmunosuppression in virus-negative inflammatory cardiomyopathy (TIMIC) trial, a randomized, double-blind, placebo-controlled single-center trial demonstrated that immunosuppression with prednisone (1 mg/kg/day) plus azathioprine (2 mg/kg/ day) improve patients’ outcome, including 6-months LVEF and hospitalization rate related to HF, in patients with virus-negative inflammatory cardiomyopathy. Thus, azathioprine has been used in recent years as first-line therapy to curb myocardial inflammation in cardiomyopathies. Steroids plus azathioprine, indeed, is now considered the standard first-line therapy to improve heart function in VNM (virus negative myocarditis), whether primary or secondary to rheumatic diseases.^[28]^

Together with prednisone as a first-line therapy, azathioprine has been used in 3 patients with endomyocardial biopsy (EBM) proven myocarditis in the same study by Pieroni *et al*, associated with a significant clinical and laboratory improvement at 12-months, with better outcomes in patients with less fibrotic changes at EMB.^[8]^ Furthermore, azathioprine has been used in 3 clinically diagnosed SSc-myocarditis described in one case report,^[6]^ and in a case series,^[18]^ and in 7 patients from a recent prospective study (Table 1). In all of these latest patients azathioprine was initiated after CMR findings suggestive for myocarditis. A follow up CMR was performed after 6 months, showing a complete normalization of T2 ratio, early gadolinium ehancement (EGE) and LGE alterations at CMR present at baseline. Consistently, improvements of cardiac symptoms and contractile function and a reduction of cardiac biomarkers were also observed.^[29]^

MMF is largely used in the management of SSc and represents a beneficial therapeutic option to curb myocardial inflammation in SSc-pHI.

The safety and efficacy of MMF has been indeed reported in heart involvement secondary to various connective tissue diseases, including SSc. Chiefly, MMF has been used in SSc-pHI both as first-line agent, or as maintenance therapy after cyclophosphamide, or as second-line therapy in patients intolerant or resistant to azathioprine, and regardless of steroid dosage.^[30]^

Recent data support the efficacy of MMF in VNM in general (prospective studies, level of evidence II^[30,31])^ and in systemic lupus erythematosus related myocarditis (case reports and a case control study ^[32,33])^; however, data on SSc-myocarditis are still limited to case series and observational studies^[4,6,23,30,34]^ (Table 1). Prospective randomized controlled trials are needed to confirm its efficacy in this clinical scenario.

Considering the safety profile and the data on clinical efficacy coming from studies assessing the usefulness of MMF in treating myocarditis, MMF should nowadays be considered the first-line therapeutic option to treat SSc-myocarditis. This is particularly true considering the presence of robust data supporting the clinical efficacy of MMF (and the better safety profile with respect to cyclophosphamide) in treating ILD, another common and severe complication of the disease, associated with an high-risk of occurrence of myocarditis.^[35]^ MMF, finally, is widely used to treat skin disease manifestations, and represents one of the therapeutic agents for SSc-related myositis.^[15,16]^

### Biologic Therapies

Data on the therapeutic use of biologic diseases modifying anti-rheumatic drugs (bDMARDs) are limited.

B-cell depletion with rituximab in SSc-pHI has been proposed. However, only two cases have been described. In one case combining oral cyclophosphamide and rituximab led to favorable results on both RVEF, LVEF and T2 mapping, while no results on T1 mapping and ECV emerged.^[23]^ In another case rituximab was used as a rescue therapy after MMF failure with only partial benefit.^[34]^

Similarly, intravenous immunoglobulins were administered in a 48-year old woman with diffuse SSc and severe cardiac involvement clinically manifested with ventricular and supraventricular arrhythmias, congestive HF and pericarditis, refractory to steroids and rituximab. The authors reported a progressive improvement of dyspnea and of heart systolic ejection fraction, paralleled by reduction of skin score.^[36]^

Recent experimental and clinical data support the pivotal role of inflammatory cytokines as interleukin (IL)-1 and IL-6 in mechanisms of heart inflammation and cardiac dysfunction in several heart diseases (including SSc), and its potential role in the inflammation-driven pathway to myocardial fibrosis. Despite a great amount of preliminary preclinical data in cardiac diseases, the therapeutic use of anti-IL1 agents (as anakinra or rilonacept) have never been reported in SSc-pHI, and no large trials to support anti-IL6 therapy with tocilizumab have been performed.^[37, 38, 39, 40, 41, 42]^

The use of tocilizumab has been recently investigated in a randomized double-blind study, and it was shown to significantly reduce the rate of lung deterioration in SSc patients.^[43]^ Moreover, SSc patients treated with tocilizumab had a lower incidence of adverse effects related to cardiac lesions: serious cardiac events were reported in 6 (5.7%) of 106 participants in the placebo group compared with 2 serious adverse events in 2 (1.8%) of 104 participants in the tocilizumab group.^[43]^

Recently, tocilizumab was used to effectively treat a patient with SSc-related myocarditis: dampening of myocardial inflammation was revealed as a reduction in myocardial edema at CMR, and by the improvement of cardiac function, clinical status and cardiac enzymes.^[44]^

Following this initial case, tocilizumab was administered in another four cases of SSc-myocarditis, with significant results in three out of four cases in terms of CMR features and contractile function.^[23,45,46]^

Thus, despite a fascinating biologic rationale, still few data are available to support the therapeutic use of bDMARDs in SSc-pHI (Table 1). However, considering the reported efficacy and safety of targeting IL-6 in SSc-ILD patients, especially in those with an “inflammatory phenotype” suggested by elevated C-reactive protein, increased platelets counts and arthritis/tenosynovitis, tocilizumab could be considered to treat pHI in SSc patients with ILD and an inflammatory phenotype. Similarly, rituximab has been widely associated with improvement of skin score and stabilization of lung function, especially in patients with early diffuse SSc.^[16]^ Thus, rituximab should be considered, even in combination with conventional DMARDs as MMF, to treat pHI in SSc patients with an early disease and a concomitant progressive skin and lung involvement.

### Haematopoietic Stem Cell Transplantation

Despite severe cardiac involvement is historically considered a contraindication for hematopoietic stem cell transplantation, there are case reports of the beneficial effects of this procedure for the treatment of SSc-related myocarditis and SSc-pHI,^[47]^ particularly with the use of less cardiotoxic protocols.^[48]^

## Drugs targeting fibrosis

Currently, there is an absence of therapeutic strategies explicitly targeting myocardial fibrosis.

The only and limited evidence available concerns the antifibrotic drug nintedanib, currently approved for the treatment of progressive SSc-ILD.

### Nintedanib

Nintedanib, a selective inhibitor of certain receptor tyrosine kinases, including vascular-endothelial growth factor receptor (VEGFR)-1–2–3, platelet-derived growth factor receptor (PDGFR)-α and β, and fibroblasts growth factor receptor (FGFR)-1–2–3, has gained recent approval for therapeutic application in SSc-ILD.^[49]^ The potential role of nintedanib to forestall fibrotic cardiac remodeling is postulated, based on the hypothesis that fibrogenic mechanisms are conserved across various etiologies, but nowadays remains purely speculative. Nintedanib attenuates macrophage activation and mitigates both vascular and fibrotic presentations in the Fra2 transgenic mouse model of SSc.^[50]^ The antifibrotic activity of the drug was correlated with an impairment in M2 monocyte polarization and a decrement in M2 macrophage populations.^[51]^ Given the observed upregulation of M2 macrophages in peripheral blood of SSc patients and the central role these cells play in myocardial remodeling and HF pathogenesis, nintedanib emerges as a compelling candidate for managing inflammation-induced fibrosis in SSc-associated cardiac pathology. A recent empirical study involving 20 consecutive patients with SSc-ILD, 10 of whom were administered nintedanib and 10 observed over time, provided support for a positive impact of anti-fibrotic treatment on SSc-pHI. There was, indeed, a significant improvement in terms of myocardial ECV abnormality as measured by CMR at the 6-month in the nintedanib cohort compared to the control group (a 1.62% decrease versus a 2.00% increase, *P* < 0.001). Additionally, the modification in RVEF was substantially different between groups, favoring the nintedanib cohort (*P* = 0.02).^[52]^

However, considering the observational nature of the study and the small sample size, these results should be taken with caution and further studies are needed to support the use of nintedanib to treat SSc-pHI.

In the next future, combining bDMARDs with conventional immunosuppressants and anti-fibrotic agents as nintedanib could be a potential therapeutic strategy to curb myocardial inflammation and myocardial fibrosis.

## Drugs targeting vasculopathy

Endothelial dysfunction and vascular abnormalities are thought to be early pathogenic events in SSc.^[25]^ In this scenario, vasodilators and anti-platelets treatment may be potentially useful in the treatment of cardiac dysfunction in patients with SSc-pHI.

### Vasodilators & Antiplatelet Agents

A reduced use of calcium-channel blockers (CCBs), widely used to treat Raynaud’s phenomenon in SSc patients, has been associated with a greater occurrence of left ventricular systolic dysfunction in the European Scleroderma Trials and Research (EUSTAR) cohort,^[53]^ thus indirectly suggesting that their use could be beneficial in preventing cardiac contractile dysfunction.

Even considering the therapeutic role of CCBs to treat peripheral vascular manifestations of the disease, their use in SSc-pHI should be considered in all patients, if not contraindicated.

The DeSScipher study highlighted the benefits of aspirin, an antiplatelet agent, and vasodilators in SSc patients. The use of vasodilators was associated with lower incidence of ventricular arrhythmias at multivariate analysis. Additionally, low-dose aspirin was associated with lower incidence of Q waves, conduction blocks and/or pacemaker implantation in univariate and multivariate analysis.^[54]^

**Figure 1:**
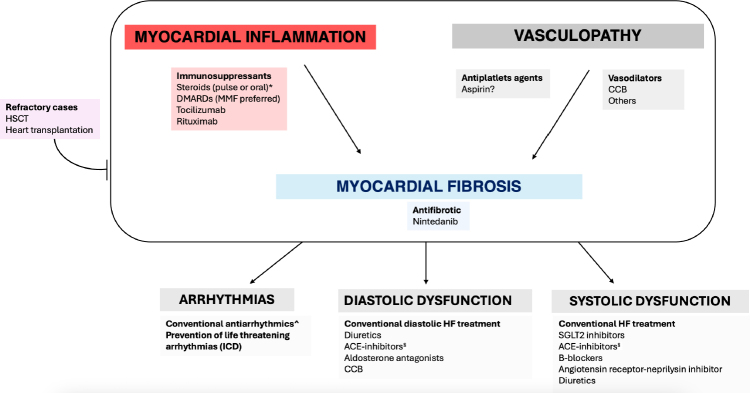
Therapeutic strategies for primary heart involvement in systemic sclerosis. Immunosuppressive treatment should be considered in any case of evidence or high suspect for myocardial inflammation. Pulse or oral corticosteroids have been successfully used in systemic sclerosis (SSc) myocarditis and pericarditis, with beneficial effects on clinical status and cardiac enzymes, but not definite benefit on systolic function. Conventional disease modifying anti-rheumatic drugs (DMARDs; azathioprine, mycophenolate mofetil [MMF], methotrexate and cyclophosphamide), as monotherapy or in combination with steroids, have shown a positive effect in SSc myocarditis. Among DMARDs, MMF is the most widely used, even considering its safety profile and tolerability, its wide use in others scleroderma organ involvement and even considering the potential cardiotoxicity of cyclophosphamide. Preliminary data on tocilizumab and rituximab are also available with positive outcomes. Calcium channel blockers (CCBs) should be considered in all SSc patients since they have been associated with a reduced risk of left ventricular systolic dysfunction in SSc patients. Limited data suggest a beneficial effects of other vasodilators to treat SSc primary heart involvement (pHI), given their potential role in treating the vascular mechanisms of the disease. The role of aspirin is still controversial. Some preliminary data are available on effectiveness of nintedanib on myocardial fibrosis at cardiac magnetic resonance. Arrhythmias and heart failure in SSc-pHI should be managed according to cardiologic guidelines^[59,63].^ *Steroids need to be used with caution in SSc patients with diffuse or rapidly-progressive disease, especially in those with anti-RNA polymerase III antibodies. ^ No data are available to recommend a specific anti-arrhythmic drug in SSc patients. § Caution must be payed using ACE-inhibitors in SSc patients at high risk for scleroderma renal crisis.

However, the occurrence of SSc-pHI despite the fact that a great percentage of SSc patients is routinely treated with CCBs and/or other vasodilator, strongly emphasize the complex and multifactorial nature of heart damage in SSc, suggesting that further research is needed to optimize treatment strategies (Figure 1). Chiefly, studies specifically addressing the efficacy of CCBs in SSc-pHI using different and more sensitive outcome measures other than reduced LVEF (which is only rarely present in SSc patients), are eagerly awaited. The therapeutic or preventive role of low-dose aspirin in SSc-pHI, furthermore, needs to be further investigated. Aspirin could indeed negatively impact on SSc-related gastro-intestinal manifestations, which are common in SSc; therefore its use should be carefully evaluated and is not routinely recommended.

## Drugs for cardiac complications & cardiologic therapy

SSc-pHI is associated with severe complications as HF, arrhythmias, and SCD; therefore, a specific treatment for this condition and prevention of life-threatening complications is fundamental (Figure 1).

### Heart Failure

Diastolic dysfunction (DD) commonly occur in SSc patients, even at early stages, as is considered one of the first markers of SSc-pHI. DD has been historically considered the consequence of myocardial fibrosis. There are few evidences on the therapeutic options for the treatment of DD in SSc. As for other forms of DD, afterload reduction can be helpful to improve cardiac function even in SSc patients.^[55,56]^

Systolic dysfunction is less common than diastolic dysfunction in SSc patients, and it could be considered as the final result of vascular damage, myocardial inflammation and fibrosis.^[11]^

The standard cardiologic therapy for systolic dysfunction includes angiotensin-converting enzyme (ACE)-inhibitors, aldosterone antagonists, diuretics and CCBs.^[55,57]^

To date, caution is needed when prescribing ACE-inhibitors to SSc patients who are at an high risk of developing scleroderma renal crisis, since a previous ACE-inhibitors therapy has been associated with a worst outcome in patient with this dramatic complication.^[58]^

More recently, the sodium-glucose co-transporter 2 (SGLT2)-inhibitors have been shown to reduce risks of clinical events in patients with HF, with early and sustained benefits regardless of ejection fraction. SGLT2-inhibitors should therefore be considered in the therapeutic algorithm for SSc-pHI manifested with HF.^[57]^

### Arrhythmias

Arrhythmias are frequent in SSc and are associated with a dismal prognosis, accounting alone for 6% of total disease-related deaths.^[59]^ It is therefore important to identify patients at higher arrhythmic risk. There are no randomized controlled trials with anti-arrhythmic drugs specifically conducted in SSc patients; therefore, the choice of therapy should be similar to that of patients without SSc,^[14]^ according to current cardiologic guidelines. In the clinical scenario of SSc, however, it is crucial to consider that β-blockers have been associated with potential negative effects on Raynaud’s phenomenon and generally on peripheral vasculopathy. However, to date, a recent study demonstrated that metoprolol, if co-administered with CCBs, may alleviate symptoms of Raynaud’s phenomenon in SSc patients.^[60]^

Besides treatment of arrhythmic burden,^[61]^ once clinically manifested, prevention of life-threatening arrhythmias and SCD is a major goal in SSc patients. In this view, patients with life-threatening arrhythmias or at high-risk for potentially severe arrhythmic manifestations, prevention of SCD with the use of an implantable cardioverter defibrillator (ICD) or invasive procedures should be encouraged. ICD insertion in high-risk SSc patients was indeed effective in reverting several episodes of ventricular tachycardia (VT) in 3 out of 10 implanted patients in a recent Italian study.^[14]^ Another study reported the efficacy of catheter ablation in a single SSc patient with ventricular tachycardia arising from right ventricular scars, suggesting ablation as a potential therapeutic strategy for treating ventricular arrhythmias in selected severe cases.^[62]^ These studies, together with others,^[5,63]^ underlie again the prognostic importance of Holter abnormalities (and electrophysiology studies in selected cases) and the clinical significance of primary prevention of SCD in selected SSc populations.

Finally, cardiac transplantation should be considered a viable option in refractory severe cases when other therapeutic strategies failed.^[64]^

## Conclusions

The prevalence of primary cardiac manifestations in SSc carries significant prognostic implications, necessitating prompt and accurate diagnostic efforts. Ischemic, inflammatory, and fibrotic pathologies represent crucial therapeutic targets. Although the heightened interest from the healthcare community, clear evidence and guidelines for effectively managing SSc-pHI are not established yet.

Immunosuppressive treatment should be considered in any case of evidence or high suspect for myocardial inflammation. Among conventional immunosuppressants, MMF represents the first therapeutic choice. Biologic therapy with tocilizumab and rituximab could be proposed in selected cases. Calcium channel blockers should be considered in all SSc patients since they have been associated with a reduced risk of left ventricular systolic dysfunction, while limited data suggest a beneficial effects of other vasodilators to treat SSc-pHI. Finally, a fascinating biologic rationale support the future potential usefulness of nintedanib to curb myocardial fibrosis.

The lack of randomized controlled trials in this disease domain, however, heightens the need for robust clinical research to guide evidence-based management. Improved understanding of the pathogenetic underpinnings of SSc-pHI, along with the creation of specific clinical recommendations that include management and therapeutic strategies for SSc-pHI, is critical to improve patient outcomes.
